# Blood pressure standards for Saudi children and adolescents

**DOI:** 10.4103/0256-4947.51787

**Published:** 2009

**Authors:** Abdullah A. Al Salloum, Mohammad I. El Mouzan, Abdullah S. Al Herbish, Ahmad A. Al Omar, Mansour M. Qurashi

**Affiliations:** a From the Department of Pediatrics, King Saud University, King Khalid University Hospital, Riyadh, Kingdom of Saudi Arabia; b From the Riyadh Children Hospital, Riyadh Medical Complex, Riyadh, Kingdom of Saudi Arabia; c From the Department of Pediatrics, Al-Yamamah Hospital, Riyadh, Kingdom of Saudi Arabia

## Abstract

**BACKGROUND AND OBJECTIVES:**

Blood pressure levels may vary in children because of genetic, ethnic and socioeconomic factors. To date, there have been no large national studies in Saudi Arabia on blood pressure in children. Therefore, we sought to establish representative blood pressure reference centiles for Saudi Arabian children and adolescents.

**SUBJECTS AND METHODS:**

We selected a sample of children and adolescents aged from birth to 18 years by multi-stage probability sampling of the Saudi population. The selected sample represented Saudi children from the whole country. Data were collected through a house-to-house survey of all selected households in all 13 regions in the country. Data were analyzed to study the distribution pattern of systolic (SBP) and diastolic blood pressure (DBP) and to develop reference values. The 90th percentile of SBP and DBP values for each age were compared with values from a Turkish and an American study.

**RESULTS:**

A total of 16 226 Saudi children and adolescents from birth to 18 years were studied. Blood pressure rose steadily with age in both boys and girls. The average annual increase in SBP was 1.66 mm Hg for boys and 1.44 mm Hg for girls. The average annual increase in DBP was 0.83 mm Hg for boys and 0.77 mm Hg for girls. DBP rose sharply in boys at the age of 18 years. Values for the 90th percentile of both SBP and DBP varied in Saudi children from their Turkish and American counterparts for all age groups.

**CONCLUSION:**

Blood pressure values in this study differed from those from other studies in developing countries and in the United States, indicating that comparison across studies is difficult and from that every population should use their own normal standards to define measured blood pressure levels in children.

The assessment of blood pressure (BP) and prevention of hypertension in children and adolescents has become a worldwide priority.^1^ Childhood blood pressure is predictive of adult blood pressure.^2^ Therefore, measurement of blood pressure is considered to be an integral part of the clinical examination.^3^ To define a standard for blood pressure in children, the Task Force on Blood Pressure Control in Children in the United States published a series of reports on blood pressure levels related to age, height and weight from birth to 18 years.^4^–^6^ The distribution of blood pressure levels and the prevalence of hypertension vary in different racial and ethnic groups.^7^–^11^ The variation is dependent upon a multitude of factors, both genetic and environmental.^4^ Based on these variations, reference norms developed for one particular population may not be applicable to others.^7^–^10^ Local reference data are essential to evaluate observed blood pressure values.

A number of epidemiological studies have established normal blood pressure values in different populations,^12^ but few are from developing countries.^7^,^8^,^13^ To our knowledge, there are no blood pressure reference data on Arab children based on a nationwide survey. This study was designed to provide age-related blood pressure reference standards for Saudi Arab children.

## METHODS

Our study was part of the Health Profile of the Saudi Arabian Children and Adolescents project, which was a house-to-house survey of 14000 randomly selected households from all provinces in Saudi Arabia, including urban and rural areas. Households were randomly selected by a multi-stage probability sampling procedure from a stratified listing based on the updated 2000-2001 census. This computerized process was performed with the assistance of the General Directorate of Statistics, Ministry of Planning, who provided details of the selected households in cities and villages, including road and street maps. A sub-sample was selected randomly from the original main sample to measure blood pressure. Workshop training of field teams was conducted in each of the 13 regions of the country. The workshops included oral presentations and small group training on procedures for locating selected households, explanations of the questionnaire, family interviews, clinical examinations of the children and the taking of measurements and recording of data. The training included practical demonstrations to members of the field teams on how to use and maintain the blood pressure measurement devices.

Specific guidelines in Arabic and English were provided to the members of the teams. Each team consisted of one physician and one to two female nurses. The clinical examination of the children and adolescents was performed by the physicians to determine eligibility for measurements. Only healthy children and adolescents as determined by interview, clinical examinations and anthropometric measurements were eligible for measurement of BP. The survey questionnaire was designed to provide basic information about the subject, including birth date, perinatal history, nutrition, childhood illnesses, socioeconomic status of the family and body measurements. The exact birth date was considered to be particularly important and acceptable only when it was completely recorded from an official document. The exact date of measurement was also noted, both dates essential for the determination of the exact age at the time of measurements.

Electronic devices using oscillometric techniques were used in the study. The devices fulfilled the American Association for the Advancement of Medical Instrumentation (AAMI), and were graded A for both systolic and diastolic pressure under the British Hypertension Society (BHS) protocol, as recommended by the European Society of Hypertension.^14^ All the devices were new and purchased especially for the study (Accutorr Plus, Datascope Corp, NJ, USA). The cuff was appropriate to the size of the upper arm according to the standard technique recommended by the working group report from the National High Blood Pressure Education Program.^4^,^5^ The right arm was used for consistency in comparison with other studies. Two readings, one at the end of the interview and the other at the end of the physical examination, were performed for each subject with an interval of 5 minutes and in the presence of both parents. For children younger than 2 years, the readings were taken in the supine position and for children older than 2 years, the readings were taken in the sitting position. The lowest of the two readings was recorded for the final analysis. A pilot study was performed to test all the components of the project before the actual start of the main study.

Data collection was performed over a period of 2 years (2004-2005) by house-to-house visits. Precautions were taken to ensure reliability and accuracy of measurements. In addition to the use of equipment known for high accuracy, intra- and inter-observer reliability were tested by selection of 1% of the children to be re-measured by the same or another observer. Multiple frequency analysis was used to detect any missing data, inconsistencies and other types of errors. All question-able data were double-checked.

The SAS system software was used. Descriptive statistics (mean, standard deviation and percentiles) were used to explore the data. Correlation and simple regression analysis were used to assess the linear relationship between two continuous variables. All values were related to the age and sex of the children. The criteria used to establish normal and abnormal were similar to those of the Second Task Force report on blood pressure control in children (normal 50th-90th percentile), (high normal 90th-95th percentile), (high >95th percentile).^4^,^5^

## RESULTS

**Figure 1:**
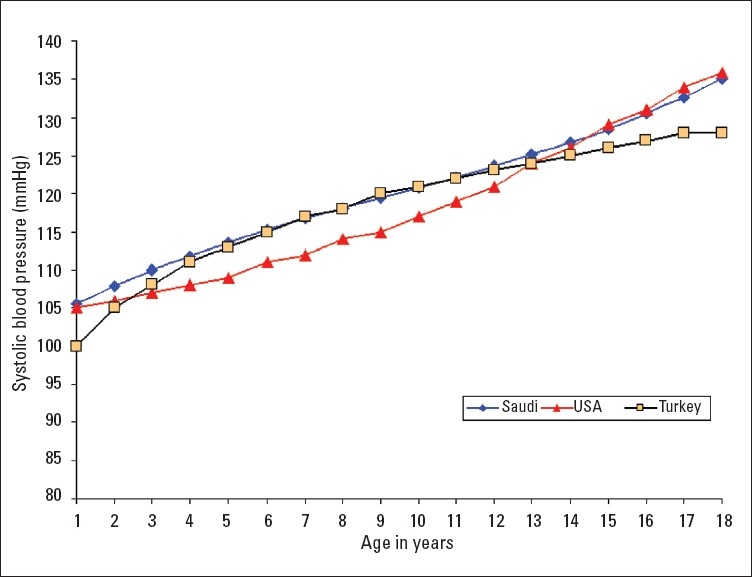
Comparison of the 90th percentile of systolic blood pressure levels of Saudi Arab boys with the values of American^4^ and Turkish^7^ boys.

**Figure 2:**
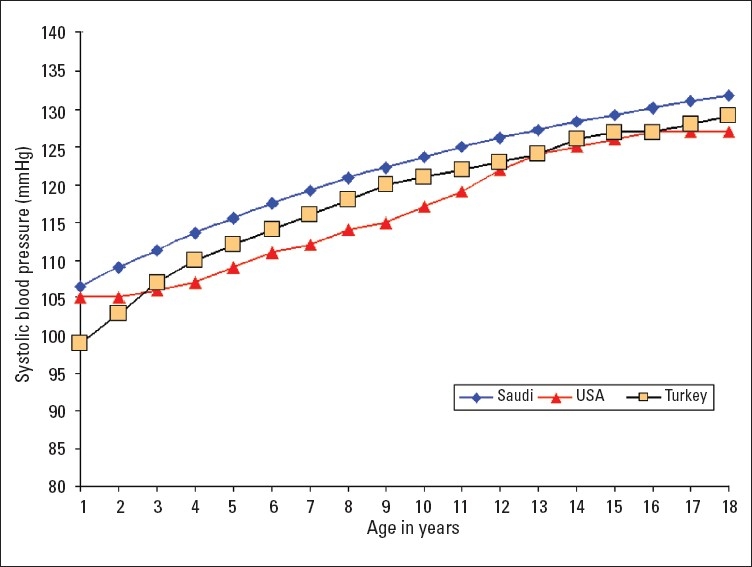
Comparison of the 90th percentile of systolic blood pressure levels of Saudi Arab girls with the values of American^4^ and Turkish^7^ girls.

**Figure 3:**
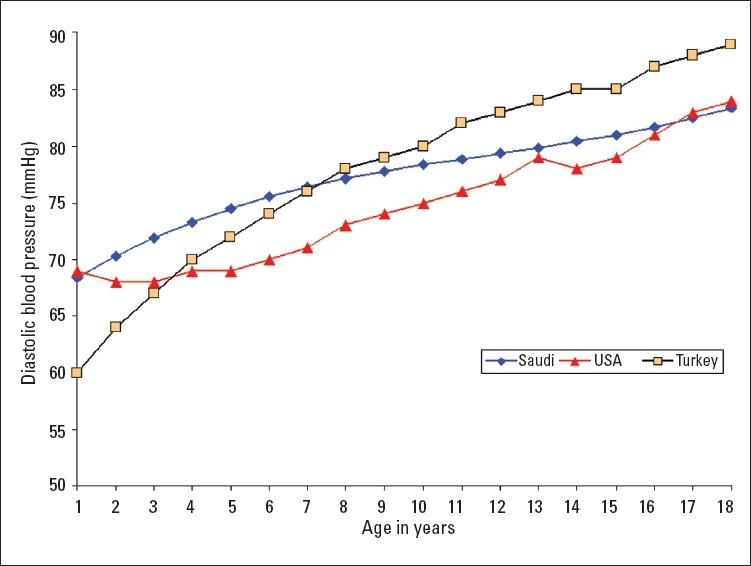
Comparison of the 90th percentile of diastolic blood pressure levels of Saudi Arab boys with the values of American^4^ and Turkish^7^ boys.

**Figure 4:**
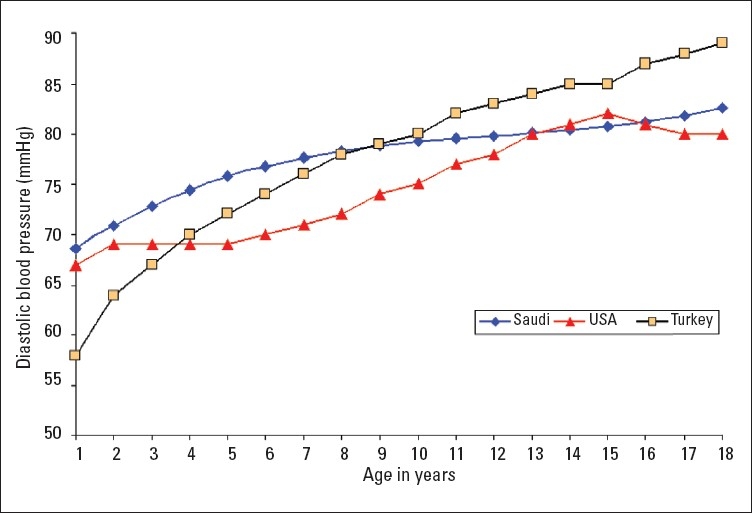
Comparison of the 90 percentile of diastolic blood pressure levels of Saudi Arab girls with the values of the American^4^ and Turkish^7^ girls.

A total of 16 226 Saudi Arabian children (7928 girls and 8298 boys) from birth to 18 years of age were examined in this study. Smooth percentile values of systolic blood pressure (SBP) and diastolic blood pressure (DBP) for these children according to age and sex are shown in Tables 1–4. SBP and DBP rose steadily with age in both boys and girls. The average annual increase in SBP for boys was 1.66 mm Hg and 1.44 mm Hg in girls (Table 1, 2). There was no significant differences in the increment in pre-pubertal and pubertal age group in boys, while in girls the SBP increment was 1.77 mm Hg up to the age of 9 years and it then decreased to 1 mm Hg/year from 10 to 18 years (Table 2). The corresponding increments were higher for girls than boys in the first 9 years of life. The average annual increases in DBP throughout childhood and adolescence for boys and girls were 0.83 mm Hg and 0.77 mm Hg, respectively (Tables 3, 4). Of note, the DBP in boys rose sharply at the age of 18 years (Table 3), which is probably related to the stress associated with this age group. The 90th percentile of SBP and DBP values in this study were compared for each age with the 90th percentile of SBP and DBP reported by one international study^4^ and one regional study^7^ (Figures 1–4). The 90th percentile of SBP in boys was closer to the Turkish levels for the age group of 5-11 years, and both are higher than the levels in the American study (Figure 1). For the ages of 14 to 18 years Saudi and American levels were significantly higher than those of the Turkish children. The 90th percentiles for SBP measurement for Saudi girls were higher than for their American and Turkish counter-parts in all age groups (Figure 2). The 90th percentiles for DBP in Saudi boys were higher than both American and Turkish children in the first 6 years of life, and then became lower than in Turkish children. By the age of 16 years the level of DBP in American and Saudi children were closer to each other, but significantly lower than the Turkish levels (Figure 3). A similar pattern was noted for DBP in girls (Figure 4).

**Table 1:** Smoothed percentiles of systolic blood pressure for boys (1-18 years).

Age (years)	Number	50th	75th	90th	95th
1	598	93	99	106	109
2	403	95	101	108	112
3	453	97	104	110	114
4	502	99	105	112	116
5	545	101	107	114	117
6	497	103	109	115	119
7	555	104	110	117	121
8	508	105	112	118	122
9	501	107	113	120	123
10	557	108	114	121	125
11	536	110	116	122	126
12	472	111	117	124	127
13	458	113	119	125	129
14	439	114	120	127	131
15	389	116	122	129	132
16	374	118	124	130	134
17	293	120	126	133	137
18	218	123	129	135	139

**Table 2:** Smoothed percentiles of systolic blood pressure for girls (1-18 years).

Age (years)	Number	50th	75th	90th	95th
1	573	93	100	106	110
2	393	95	102	109	113
3	468	98	105	111	115
4	476	100	107	114	117
5	495	102	109	116	119
6	469	104	111	117	121
7	540	105	113	119	123
8	474	107	114	121	125
9	498	109	116	122	126
10	524	110	117	124	128
11	451	111	118	125	129
12	437	112	120	126	130
13	422	113	121	127	131
14	439	114	122	128	132
15	364	115	123	129	133
16	352	116	124	130	134
17	309	117	124	131	135
18	244	118	125	132	136

**Table 3:** Smoothed percentiles of diastolic blood pressure for boys (1-18 years).

Age (years)	Number	50th	75th	90th	95th
1	598	57	63	68	72
2	403	59	65	70	74
3	453	60	66	72	75
4	502	62	68	73	77
5	545	63	69	74	78
6	497	64	70	76	79
7	555	65	71	76	80
8	508	66	72	77	80
9	501	66	72	78	81
10	557	67	73	78	82
11	536	67	73	79	82
12	472	68	74	79	83
13	458	68	74	80	83
14	439	69	75	80	84
15	389	70	76	81	84
16	374	70	76	82	85
17	293	71	77	82	86
18	218	72	78	83	89

**Table 4:** Smoothed percentiles of diastolic blood pressure for girls (1-18 years).

Age (years)	Number	50th	75th	90th	95th
1	573	57	63	69	72
2	393	59	65	71	74
3	468	61	67	73	76
4	476	63	69	74	78
5	495	64	70	76	79
6	469	65	71	77	80
7	540	66	72	78	81
8	474	67	73	78	82
9	498	67	73	79	82
10	524	68	74	79	83
11	451	68	74	80	83
12	437	68	74	80	83
13	422	69	75	80	83
14	439	69	75	80	84
15	364	69	75	81	84
16	352	70	76	81	85
17	309	70	76	82	85
18	244	71	77	83	86

## DISCUSSION

The incorporation of blood pressure measurement into the routine pediatric examination as well as the publication of national norms for blood pressure in children not only enables detection of significant asymptomatic hypertension secondary to a previously undetected disorder, but also confirms that mild elevation in blood pressure during childhood is more common than previously recognized, particularly in adolescents.^2^,^6^ It is now understood that hypertension detected in some children may be a sign of an underlying disease, such as renal parenchymal disease, whereas in other cases elevated blood pressure may represent the early onset of essential hypertension.^5^

The standard blood pressure percentile tables for children and adolescents provide arterial pressure values frequently seen in a large numbers of subjects presumed to be normal. Reference norms developed for one particular population may not be applicable to another because of racial, ethnical and cultural differences across the world.^15^ The local reference data is essential to evaluate any observed blood pressure values. A number of epidemiological studies have established normal blood pressure values in different populations.^12^ Combined data from several studies on references for blood pressure have been published from studies performed in the United States^4^–^6^ and Europe.^16^ The blood pressure percentiles presented here are based on data collected using a consistent and rigorous method in a representative sample of 16 226 children and young people living in Saudi Arabia.

The definition of normal blood pressure values in children is based on mercury sphygmomanometry.^4^–^6^ Accurate blood pressure measurement by mercury sphygmomanometry is particularly difficult in children because of widespread misinterpretations of the Korotkov sounds.^4^,^6^,^15^,^17^–^19^ To overcome this problem and to eliminate human error, we used automated oscillometric devices, which represent a relatively new technology for blood pressure measurement.^20^ The device is easy to use with small children because there is no need for auscultations.^20^,^21^ Oscillometry is widely accepted in hospitals. Both systolic and diastolic pressures are calculated from measured mean artrial pressure in a sufficiently accurate manner.^20^ These devices continue to gain wider use.^20^ Earlier oscillometric devices often overestimated blood pressure compared with mercury sphygmomanometry, but newer models produce estimations of blood pressure that are very close to those of mercury sphygmomanometry,^15^ particularly after the introduction of validation protocols by the American Association for the Advancement of Medical Instrumentation (AAMI) and the British Hypertension Society (BHS).^20^ Some investigators have suggested that oscillometric devices might be superior to the auscultatory method, particularly in children, as a result of increased accuracy, reduced variability and ease of use.^12^,^15^,^20^,^22^ Mercury sphygmomanometry, although conventionally regarded as the reference method, has inherent variability as a result of technique and human error, which has not been assessed comprehensively.^15^ For optimal results, we used oscillometric devices that fulfill the AAMI criteria and are graded A for both SBP and DBP under the BHS protocol, and recommended by the European Society of Hypertension.^14^

Clinic blood pressure measurements tend to be higher than home measurements,^23^ a phenomenon known as “white coat hypertension”.^15^ To record the real normal blood pressure for the subject by eliminating the effect of the “white coat”, the measurement should be obtained in “normal circumstances” for the subject. We believe that the normal circumstances for the child is the home, which is certainly more comfortable to the child than the health hall at the school or the medical center. Most of the studies from which the working group Task Force derived their data are from a single measurement.^4^ The first (or single) readings are usually higher than the average of multiple readings.^20^ The average of multiple blood pressure readings is closer to basal blood pressure levels.^5^ In our stud,y blood pressure measurements were based on the lowest of two measurements within five minute intervals. We believe that there is no need for additional effort to obtain more than two readings, once the child realizes, with the support of his family, the painless and benign nature of the procedure.

It is of interest to compare our results statistically with those of other studies in other developing countries or in the United States, but it should be noted that the comparison of blood pressure values across these studies is difficult, since different criteria were employed.^4^,^7^,^8^ In conclusion, these data on blood pressure measurements are the most recent, comprehensive and representative of the Saudi Arab population of children and adolescents. To our knowledge, this is the biggest prospective study of blood pressure in one of the developing countries. This reference data should help practicing clinicians in a better assessment of their patients blood pressure than the respective standards of other populations.
